# “Pigtail through snare” technique: an easy and fast way to retrieve a catheter fragment with inaccessible ends

**DOI:** 10.1186/s42155-021-00218-6

**Published:** 2021-03-02

**Authors:** Kensaku Mori, Chika Somagawa, Shun Kagaya, Masafumi Sakai, Satoshi Homma, Takahito Nakajima

**Affiliations:** 1 Department of Radiology, Faculty of Medicine, University of Tsukuba, 1-1-1 Tennodai, Tsukuba, 305-8575 Japan; 2 Department of Radiology, University of Tsukuba Hospital, 2-1-1 Amakubo, Tsukuba, 305-8576 Japan; 3 Department of Cardiology, Faculty of Medicine, University of Tsukuba, 1-1-1 Tennodai, Tsukuba, 305-8575 Japan

**Keywords:** Pigtail catheter, Snare catheter, Central venous catheter, Foreign body, Retrieval

## Abstract

**Background:**

A catheter fragment with inaccessible ends can be retrieved using the well-known two-step method: making a free end with a pigtail catheter and seizing it with a snare catheter. Here we propose an easier and faster modification, named the “pigtail through snare” technique.

**Case presentation:**

A 61-year-old female patient underwent removal of a central venous catheter fragment migrating to the right atrium. Both ends located in the right atrial appendage and left hepatic vein were inaccessible. Initially, a snare loop was opened in the inferior vena cava and a pigtail catheter was advanced through the snare loop to hook the catheter fragment. The free end was created by pulling the pigtail catheter, dragged automatically into the snare loop, grasped, and retrieved immediately.

**Conclusions:**

By passing the pigtail catheter through the snare loop in advance, the snaring maneuver becomes easy and fast in retrieving the catheter fragment with inaccessible ends.

## Background

Migration of catheter fragments caused by the creation of central venous access is a rare but severe complication. The percutaneous transluminal approach is widely used for the retrieval of these foreign bodies. A snare loop catheter is the main device for this purpose; however, it is necessary to perform the following two-step method when there are no accessible free ends on the catheter fragments. Initially, a pigtail catheter is advanced to hook or to entangle the catheter fragments and pulled to make an accessible free end. Subsequently, a snare catheter is advanced to the targeted free end and is used to tighten the catheter fragment to retrieve Here we propose an easier and faster modification of the two-step method, named the “pigtail through snare” technique.

## Case presentation

**Fig. 1:**
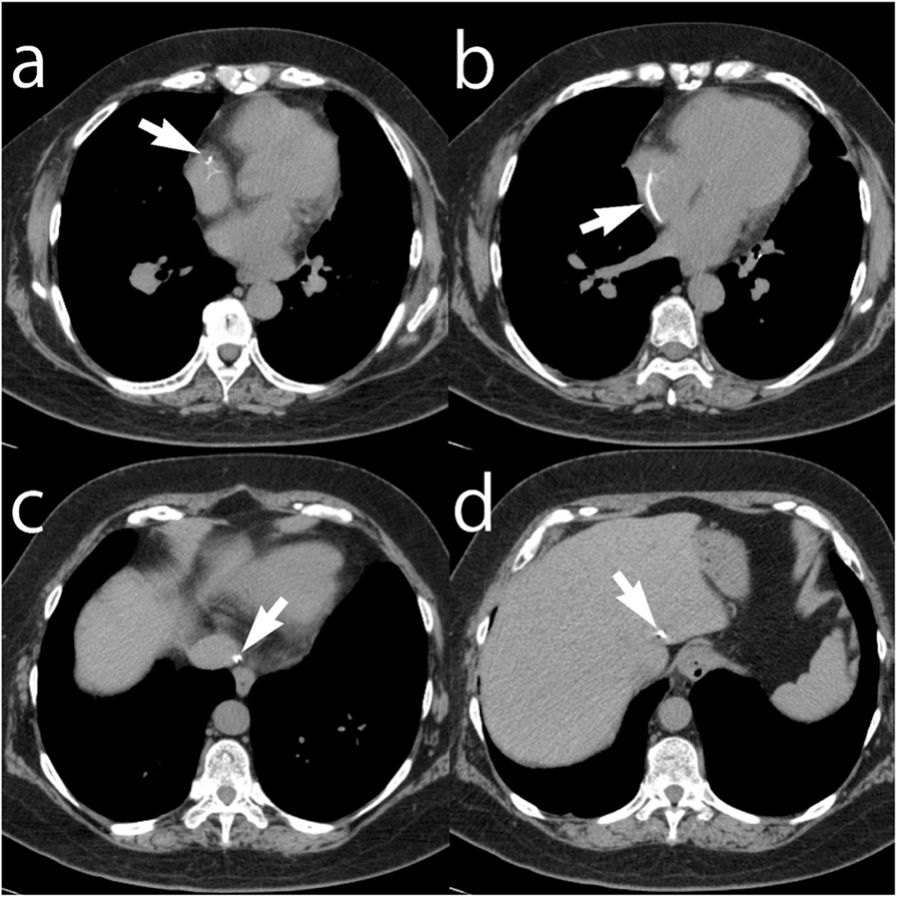
Non-contrast-enhanced CT images in a 61-year-old female patient with colon cancer show a dislodged central venous catheter fragment with inaccessible ends. The catheter was implanted 16 years ago to treat breast cancer and its fragment was migrated in the right atrium at least 3.5 years ago. **a** The upper end of the catheter fragment is located in the right atrial appendage. **b** It runs through the right atrium and **c** inferior vena cava until **d** its lower end strayed into the left hepatic vein

A 61-year-old female patient was referred to our hospital to undergo surgical resection of colon cancer. She underwent bilateral mastectomy for breast cancer and implantation of a central venous port system via the left subclavian vein for chemotherapy 16 years ago in another institution. In addition, she has been suffering from pulmonary hypertension for 10 years. At the preoperative workup, a chest radiograph revealed a broken central venous catheter fragment in the heart. According to a retrospective review of the chest radiographs, disruption of the catheter occurred at least 3.5 years ago. The catheter fragment was found to be lodged mainly in the right atrium on computed tomography (CT), and both ends located in the right atrial appendage and the left hepatic vein were inaccessible (Fig. 1).

**Fig. 2:**
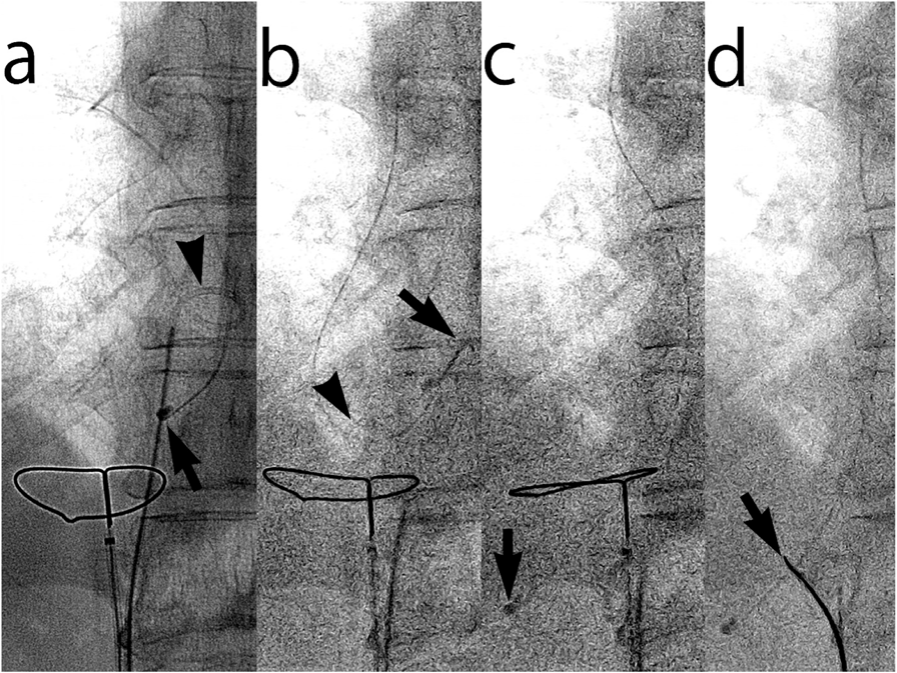
Fluoroscopic images during the retrieval of a central venous catheter fragment with inaccessible ends in a 61-year-old female patient with colon cancer. The catheter was implanted 16 years ago to treat breast cancer and its fragment migrated to the right atrium for at least 3.5 years. **a** The lower end of the catheter fragment is located in the left hepatic vein (arrow). The pigtail catheter (arrowhead) is advanced through the snare loop and hooked to the catheter fragment. **b** The lower end of the catheter fragment is disengaged (arrow) by pulling the pigtail catheter (arrowhead). **c** The pigtail catheter is pulled further to drag the lower end of the catheter fragment inferiorly through the snare loop (arrow). **d** The snare loop tightens the catheter fragment immediately (arrow)

Percutaneous retrieval of the catheter fragment was performed as follows: under local anesthesia, the right femoral vein was punctured and a 10-Fr sheath 25 cm in length (Supersheath®, Medikit Co. Ltd., Tokyo, Japan) was placed in the inferior vena cava (IVC). A 6-F snare catheter with a 25-mm-diameter loop (Amplatz Gooseneck Snare™, Medtronic, Minneapolis, MN, USA) was advanced into the hepatic segment of the IVC and opened in advance. Then, an angled 4-F pigtail catheter (Optiflash®, Terumo Co., Tokyo, Japan) was inserted over a 0.035-in guidewire (Radifocus®, Terumo Co., Tokyo, Japan) into the sheath side-by-side with the snare catheter. The pigtail catheter was advanced into the right atrium passing through the snare loop under multi-directional fluoroscopic guidance (Fig. 2a). The catheter fragment was then hooked and pulled inferiorly by the pigtail catheter (Fig. 2b). As a result, the inferior end of the catheter fragment disengaged from the left hepatic vein and fell into the IVC through the snare loop (Fig. 2c). Instantly, the snare loop tightened the catheter fragment at the initial position (Fig. 2d). Repositioning of the snare catheter was not necessary throughout the procedure. When the catheter fragment was being removed from the body, the pigtail catheter was first pulled into the sheath to ensure sufficient clearance to pull the folded catheter fragment into the sheath. The administration of local anesthesia to the withdrawal of the sheath took 26 min. Fluoroscopy time was 4.6 min. The dose area product was 8640 mGycm^2^ and air kerma was 68.12 mGy.

## Discussion

Central venous catheter fragments should be retrieved regardless of how long it has been dislodged due to the risk of serious reported complications such as arrhythmia, perforation, clotting, infections and even death (Fisher and Ferreyro 1978). In our case, the catheter fragment had migrated to the right atrium at least 3.5 years ago. This was possibly related to the development of pulmonary hypertension as the cause of chronic thrombogenesis. Espiritu et al. reported a similar case of pulmonary hypertension due to a retained implantable venous access device fragment in the pulmonary artery (Espiritu and Stolar 2007).

The two-step technique using a pigtail and a snare loop catheter in retrieving a dislodged catheter fragment is the classical method that was first reported by Greenfield et al. in 1978. When there are no accessible free ends on migrated catheter fragments, the pigtail catheter is necessary to make at least one end free that can be subsequently grasped by the snare loop catheter. Many investigators have reported the usefulness of the two-step method (Pittiruti et al. 2000; Bessoud et al. 2003; Rodrigues et al. 2007; Chuang et al. 2011; Wang et al. 2015; Pandey et al. 2018). According to the most extensive case series reported by Bessoud et al. (2003), 95 of 100 fractured and embolized central venous catheters were successfully retrieved using pigtail catheters to reposition catheter fragments before snaring in 42 cases. Chuang et al. reported that the dislodged port catheters in 23 cases were successfully retrieved with concurrent use of a pigtail and a snare catheter through a single sheath (Chuang et al. 2011). Chuang et al. raised a problem regarding the two-step method: even once the free end of the catheter fragment was created, it sometimes passed into the heart, and it would become inaccessible again before or during the snaring maneuver. Therefore, they recommended that the pigtail catheter be held still after the free end was created, with the snare catheter grasping either the free end of the catheter fragment or the tip of the pigtail catheter. Recently, Haga et al. modified the two-step method in case of a catheter fragment with inaccessible ends located in the right atrium and ventricle (Haga and Shindo 2020). A 0.035-in. guidewire was inserted into the right ventricle, crossed with the catheter fragment, and returned to the right atrium, where a snare grasped it.

**Fig. 3:**
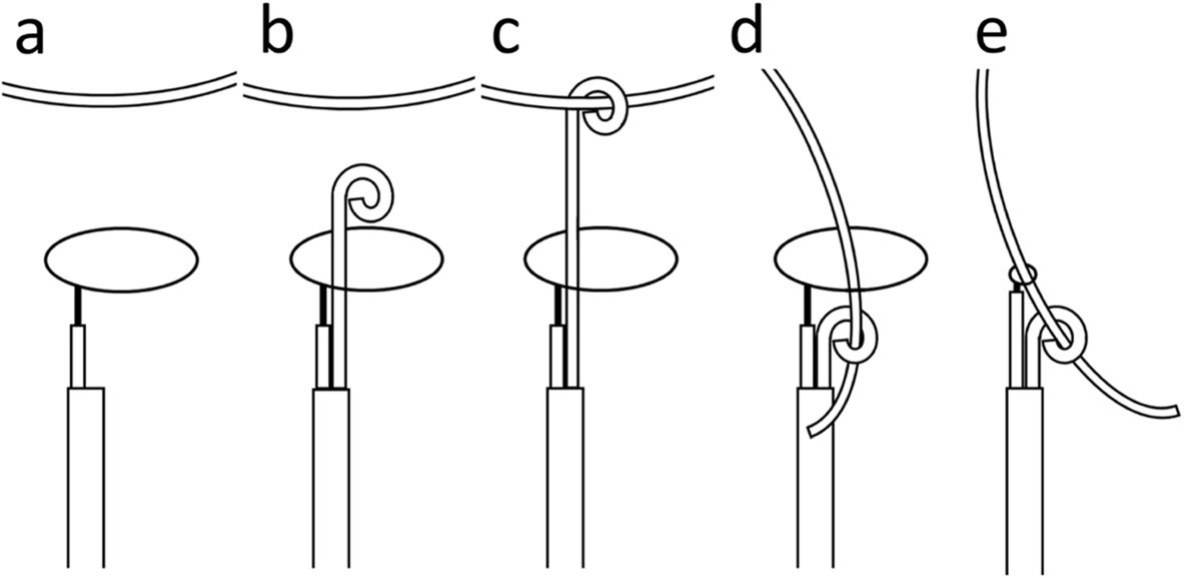
Schematic drawing of the “pigtail through snare” technique. **a** A snare loop initially opens near the target catheter fragment with inaccessible ends. **b** A pigtail catheter passes through the snare loop in advance. **c** The pigtail catheter hooks the catheter fragment. **d** The pigtail catheter pulls the catheter fragment that falls automatically into the snare loop. **e** The snare loop grasps the catheter fragment at the initial position

In the present case, we further modified the two-step method called the “pigtail through snare” technique (Fig. 3). In this modified method, the free end of the catheter fragment is pulled into the snare loop by the pigtail catheter that has been passed through the snare loop in advance. This is easier and faster than the conventional two-step method because of the following reasons. First, it is easier to pass the pigtail catheter through the snare loop in advance than to catch the free ends of the catheter fragment by controlling the snare catheter. Second, the time consumed for the snaring maneuver is almost eliminated. The single-puncture approach using a relatively thin 10-F sheath is another advantage of this method. On the contrary, this technique has two limitations. First, this is a single case report. Thus, further investigation is necessary whether the technique is feasible in another place, including the superior vena cava, right ventricle, and pulmonary arteries. Second, two operators in charge of pigtail and snare catheters are desirable for quickly grasping the created free end of the catheter fragment without missing it.

## Conclusion

Even in long-standing cases, dislodged catheter fragments should be retrieved. When a pigtail and a snare loop catheter are used simultaneously to retrieve the catheter fragment with inaccessible free ends, the procedure will become easier and faster by the “pigtail through snare” technique.

## Data Availability

Not applicable.
